# Market prediction using machine learning based on social media specific features

**DOI:** 10.1007/s10015-023-00857-z

**Published:** 2023-02-22

**Authors:** Satoshi Sekioka, Ryo Hatano, Hiroyuki Nishiyama

**Affiliations:** Department of Industrial Administration, Graduate School of Science and Technology, Tokyo University of Science, 2641 Yamazaki, Noda Chiba, Japan

**Keywords:** Machine learning, Natural language processing, Cryptocurrency

## Abstract

In recent years, unspecified messages posted on social media have significantly affected the price fluctuations of online-traded products, such as stocks and virtual currencies. In this study, we investigate whether information on Twitter and natural language expressions in tweets can be used as features for predicting market information, such as price changes in virtual currencies and sudden price changes. Our method is based on features created using Sentence-BERT for tweet data. These features were used to train the light-gradient boosting machine (LightGBM), a variant of the gradient boosting ensemble framework that uses tree-based machine learning models, with the target variable being a sudden change in closing price (sudden drop, sudden rise, or no sudden change). We set up a classification task with three labels using the features created by the proposed method for prediction. We compared the prediction results with and without these new features and discussed the advantages of linguistic features for predicting changes in cryptocurrency trends.

## Introduction

**Fig. 1:**
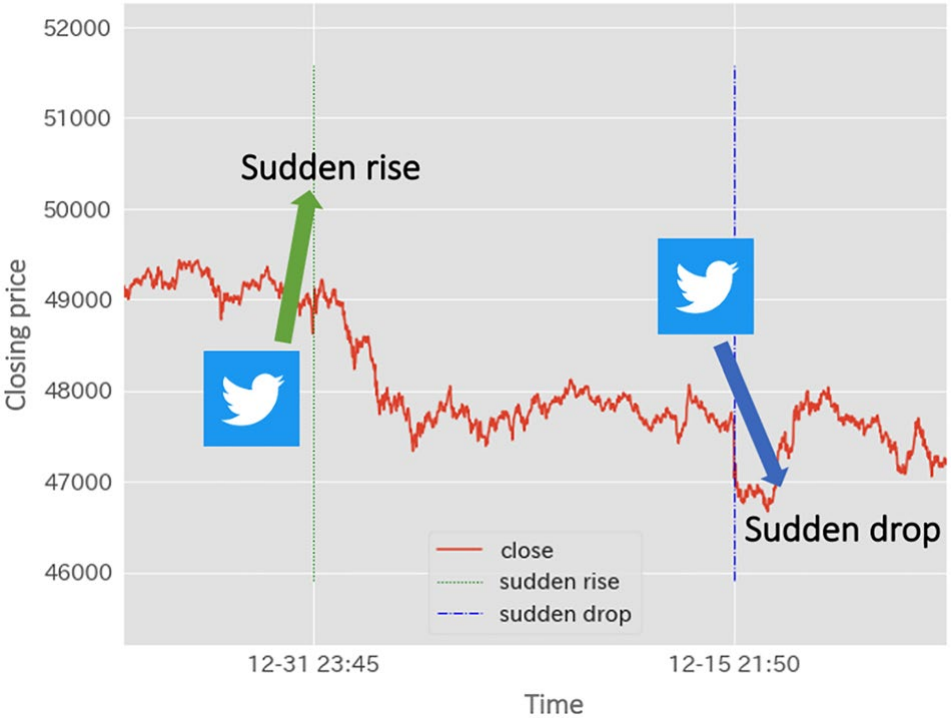
Using social media features to predict sudden price changes

In recent years, texts posted on social media have significantly affected price fluctuations in online trading, such as stock trading and cryptocurrency exchanges. Therefore, the use of big data obtained from social media has attracted considerable attention for the observation and prediction of real-world events. Taking Twitter as a typical example, the price of a cryptocurrency fluctuated significantly immediately after a tweet was posted by Elon Musk on May 14, 2021. One of the main reasons for this phenomenon is that social media interactions among many people, including super-influencers and traders, significantly impact cryptocurrency prices. Additionally, cryptocurrency price trends are easily influenced by investor sentiments. Based on this observation and the hypothesis that social media actuates real-world events, we expect that data regarding linguistic expressions on social media might be helpful for market prediction. Therefore, the objective of this study is to investigate how texts posted on Twitter affect the price volatility of cryptocurrencies traded online, such as Bitcoin (BTC) and Tether (USDT). Note that we have not restricted our attention to texts posted by famous influencers such as Elon Musk.

In particular, we show that information on natural language expressions in tweets on Twitter and tweet-specific information, such as the number of relevant tweets per minute, is helpful for estimating changes in the prices of cryptocurrencies or stocks. To achieve this goal, we propose a machine learning method to predict sudden changes in the closing price of a cryptocurrency using both linguistic features generated by Sentence-BERT [1] and features of tweet-specific information obtained from social media. Figure 1 shows an example of using tweet data to predict a sudden change in price (closing price) that could not be predicted without using tweet data in this study.

The outline of the proposed method is as follows. We first extracted BTC to USDT (BTC/USDT) information in the form of a time bar per minute from Binance, a virtual currency exchange. This was used to construct a dataset that included the opening price, closing price, high price, low price, and BTC/USDT trading volume. Next, we used the Twitter API to collect information on the tweets. From this, we created both linguistic features using Sentence-BERT and tweet-specific features and aggregated them using time bar intervals. We were able to extract more linguistic features in this manner than by inputting sentences directly into BERT [2].

Tweet-specific features included the number of tweets per minute, number of favors and retweets, and features obtained from text without Sentence-BERT, such as the length of the text and number of emojis in a tweet. Subsequently, we designed a classification task with three labels regarding the BTC-USDT closing price, corresponding to a sudden drop, sudden rise, or neither, where the latter signified a normal trend. We associated these labels with the corresponding features to create a dataset. We then trained the machine learning model using the light-gradient boosting machine (LightGBM) [3]. This method allowed us to predict sudden changes in the closing prices of cryptocurrencies. To discuss the advantages of using linguistic features to predict changes in cryptocurrency trends, we compared the performance of our models with and without the features generated by the proposed method.

## Literature review

Caron et al. [4] used a BERT-based model to make regression predictions of stock return volatility from textual information found in the management discussion and analysis (MD &A) section of a 10-K annual accounting report and evaluated the results using the mean absolute error (MAE). They made predictions based on hard information (i.e., numerical data with clearly defined meanings) and soft information (i.e., data expressed in texts such as corporate reports, press releases, and news articles). The best performance was obtained by predictions based on the BERT-based model (soft information) and the average historical volatility (hard information). The performance was better when trained only on the data from recent years.

Ohana et al. [5] predicted a major price drop in the S &P 500 using the LightGBM [3] approach with a set of over 150 technical, fundamental, and macroeconomic variables. The results showed that Shapley additive explanation (SHAP) values [6] robustly identified the most important variables that predict stock market crises, which helped explain the crisis probability for each date. The authors were also able to predict the S &P 500 financial crash of March 2020 by increasing the crash probability to 27% on February 3, 2008, and by sharply increasing the crash probability to 61% at the start of the COVID-19 pandemic on March 2, 2020.

Mehta et al. [7] sought to determine the correlation between the movement of a company’s stock prices and its public opinion. They also conducted sentiment analysis of social media data to enhance stock market predictions using machine learning.

In this study, we collected information from social media, as described by Mehta et al. [7]. We created a natural language model, similar to that of Caron et al. [4], to predict sudden price changes in commodities traded online, as in Ohana et al. [5]. Hence, we attempt to predict sudden price changes that would not otherwise be possible without the use of social media.

## Proposed method and implementation

**Fig. 2:**
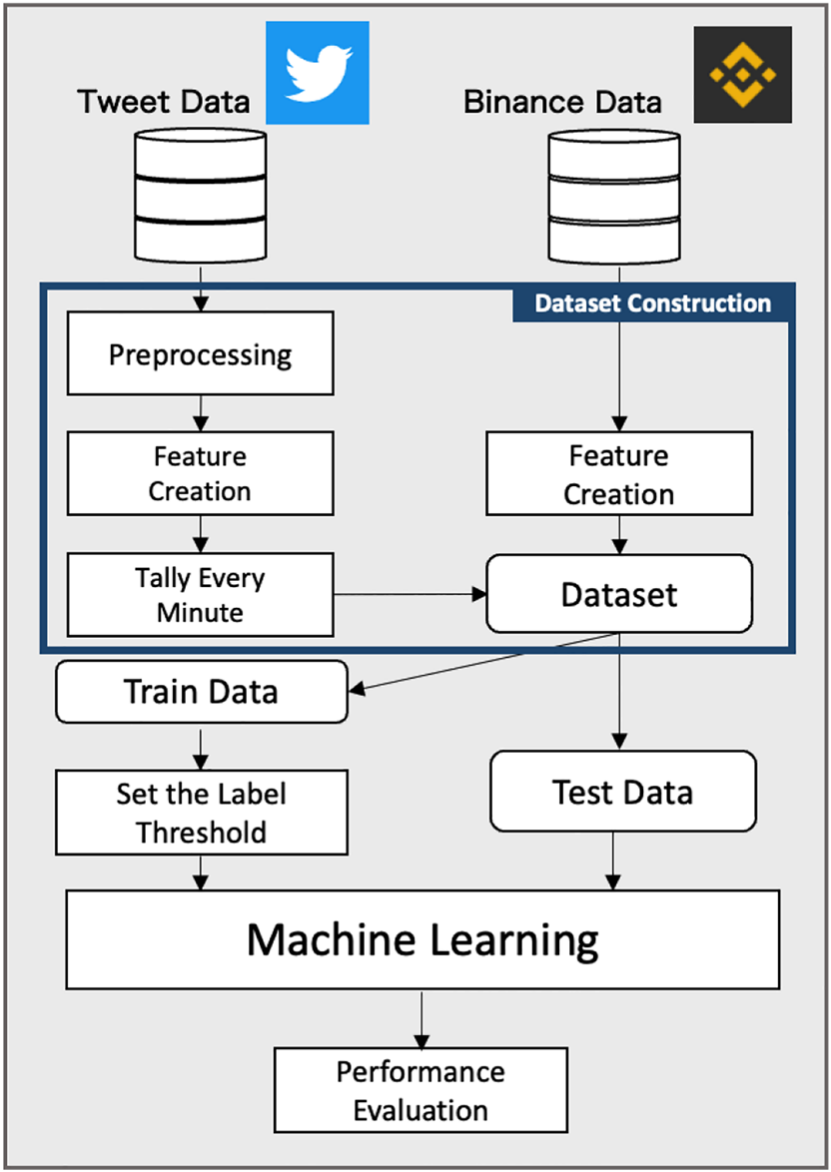
Overview of the proposed method

**Fig. 3:**
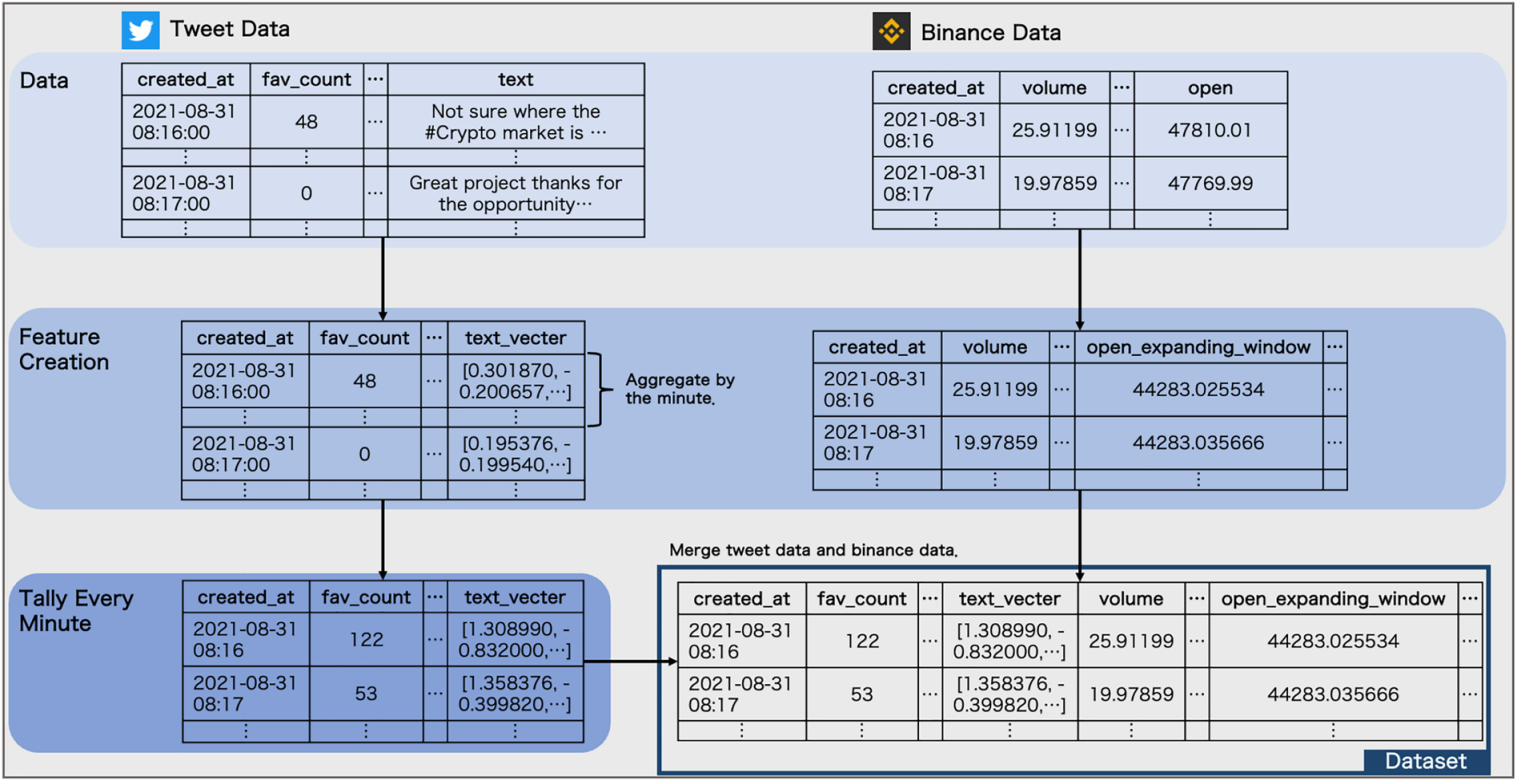
Details of the dataset construction

An overview of the proposed method is presented in Fig. 2. The outline of the procedure is as follows. Using the Twitter API and Binance API, we obtained BTC/USDT tweet data and time bar information in 1 min increments. The time-bar information is then marked using the closing price difference. A threshold was obtained to label a certain percentage of sudden price changes based only on the training data (i.e., more than 75% of the difference in the closing prices was considered as a sudden increase, less than 25% was a sudden decrease, and neither case was considered to be normal). We also formatted the tweet data into a distributed representation using Sentence-BERT. The natural language data were aggregated every minute, as explained in the time bar. Afterward, we trained a machine learning model to classify the changes in closing price with the three labels of sudden rise, sudden drop, or neither, and evaluated the obtained model.

### Collecting tweet and bincance data

Our raw dataset consisted of Twitter and Binance data. We collected data in the following manner.

Twitter dataset: In this study, only English tweets that contained “BTC/USD” or synonymous words were collected. The Twitter API was used for data collection. The free version of the Twitter API is limited in that it can only retrieve data up to the previous seven days. We collected Twitter data for almost half a year using this API. In particular, we used 534,155 texts for this study. The smallest text consisted of one word and the largest of 82 words. Among the above, the first quartile was 7 words, the median was 15 words, and the third quartile was 27 words.
Binance dataset: The API provided by Binance, a virtual currency exchange, was used to obtain a time bar of BTC/USDT in Binance in increments of 1 min. The time bar is often used to represent stock prices and is a form of data sampled and organized according to the market’s transaction history, with the data separated by a certain amount of time.

Note that there were no flaws in the data that could be retrieved from the Twitter and Binance APIs. Therefore, we did not process any of the data at this stage.

### Preprocessing for tweet data

Before creating features from the acquired tweets, the text data of the collected tweets were cleaned. The contents of the formatting are as follows:

- Converting pictograms to strings.
- Deleting hashtags, images, and mentions.
- Replacing URL “https://...” with the term “url”.

### Feature creation

**Table 1:** List of extracted features

Data source	Features
Twitter	Aggregate embedding(Sentence-BERT), retweets, number of favorites, possibly sensitive, text length, length of the text after preprocessing, number of pictograms
Binance	Volume, number of trades, quote asset volume, base asset volume, expanding window features, high, open, low

($\divideontimes$:Sensitivity is a value indicating whether the content is sensitive or not)

In Fig. 3, we show the details of feature creation from both (preprocessed) Tweet data and Binance data and in Table 1, we indicate which features were obtained. For Binance data, we created new features for the columns representing prices other than close, such as open, high, and low, for all past periods. In what follows, we call them features of “expanding window”. As for the other values from the Binance data, we used them as features directly to avoid any leakage (see also Table 1, the row of features from the Binance data).

For the preprocessed text data of tweets, we obtained a 768-dimensional variance matrix using a pretrained model called all-mpnet-base-v2 based on the Sentence-BERT [1] architecture provided by Hugging Face. In contrast to the traditional BERT approach, Sentence-BERT uses Siamese and triplet networks to fine-tune a pre-trained model.

The distributed representation created by Sentence-BERT and other Twitter specific information, such as favors and retweets, was aggregated per minute. Hence, we could reflect feature information that would be truncated if there were many tweets per minute, as BERT cannot contain more than 512 tokens because of its structural limitations. Later, we combined the processed Tweet data with the Binance data based on their time.

### Partitioning the dataset

To handle the time-series data appropriately, we split the training and test data without shuffling.

Additionally, to demonstrate the usefulness of features based on natural language expressions, we removed the range of data that corresponded to no tweet data from the dataset.

### Assigning sudden price changing labels based on threshold values

In this study, we constructed a multiclass classification model of changes in closing prices of Binance data, such as sudden rise, sudden drop, and neither. To avoid leakage, we calculated the changes in the closing price of the Binance data in the training dataset and stored the thresholds to determine the values of sudden changes. First, we calculated the change ${\varDelta }p$ in closing price from *i* to $i+1$ as follows:

1 $$\begin{aligned} {{\varDelta }p_\mathrm {close_{i}} = \frac{p_\mathrm {close^{(i+1)}}-p_\mathrm {close^{(i)}}}{p_\mathrm {close^{(i)}}},} \end{aligned}$$

where $p_\mathrm {close^{(i)}}$ shows the close price of *i*.

We then stored values greater than 75% and less than 25% of the magnitude of the change in the closing price of the test data as threshold values. We set the thresholds for the present study to 25% and 75% to avoid constructing an imbalanced dataset. Of course, these thresholds can be changed according to the demand for real operation. Finally, we assigned a sudden change label to the sample corresponding to a sudden change (sudden rise or drop) in the test and training datasets, respectively.

### Classification by machine learning

We used the LightGBM [3], which is a variant of the gradient-boosting ensemble framework that uses tree-based models in supervised machine learning. The trained model can robustly classify an objective variable according to the explanatory variables.

We fixed our model to the LightGBM and conducted experiments. We fixed the model for the following reasons. First, we intended to demonstrate the superiority of the information of natural language representation in tweet data. Second, the implementation of the model allowed us to specify the computation environment (GPU) easily and deal with the missing values, which are different from those of other similar tree-based ensemble frameworks such as XGBoost and random forest.

**Table 2:** Results by the combination of features and duration of data collection

	ACC (baseline)	ACC	Macro F1 value
Twitter features (train data: 2021/6/29–2021/11/9, test data: 2021/11/9–2022/1/3)	48.9	49.1	23.2
Binance features (train data: 2021/6/29–2021/11/9, test data: 2021/11/9–2022/1/3)	48.9	50.0	27.9
Twitter features $+$ Binance features (train data: 2021/6/29–2021/11/9, test data: 2021/11/9–2022/1/3)	48.9	50.0	**28**.**3**
Twitter features (train data: 2021/6/29–2021/11/27, test data: 2021/11/27–2022/1/3)	49.2	49.3	22.9
Binance features (train data: 2021/6/29–2021/11/27, test data: 2021/11/27–2022/1/3)	49.2	50.0	28.7
Twitter features $+$ Binance features (train data: 2021/6/29–2021/11/27, test data: 2021/11/27–2022/1/3)	49.2	50.0	**29**.**3**
Twitter features (train data: 2021/6/29–2021/12/15, test data: 2021/12/15–2022/1/3)	56.8	56.8	24.6
Binance features (train data: 2021/6/29–2021/12/15, test data: 2021/12/15–2022/1/3)	56.8	56.9	25.4
Twitter features $+$ Binance features (train data: 2021/6/29–2021/12/15, test data: 2021/12/15–2022/1/3)	56.8	56.9	**29**.**6**

### Parameters

In Table 3, we list some hyper-parameters that we considered in this study for LightGBM, where the names of the hyper-parameters and the values come from a library we used [8]. As for the hyper-parameters not listed in Table 3, we used default values provided by the library. To handle the multi-class classification task, we changed the values corresponding to the hyper-parameters of “objective”, “num_class” and “metric.” We also adjusted the values corresponding to the hyper-parameters of “num_iteration”, “device”, and “seed” to obtain suitable results, according to several trials of our experiment, which will be described in the next section.

**Table 3:** Changed hyper-parameters for LightGBM

Parameter	Value
Objective	Multiclass
Num_class	3
Metric	Multi_error$^{1}$
Num_iteration	1000
Device	gpu
Seed	42

$^{1}$ “multi_error” means the error rate for multi-class classification

For BERT, we used exactly the same parameters as in the pre-trained model called “roberta-base-nli-stsb-mean-tokens”. This is because we evaluated the performance of “roberta-base-nli-stsb-mean-tokens”, “finbert”, and other pre-trained models during our preliminary experiment and eventually obtained the best embedding results using “roberta-base-nli-stsb-mean-tokens”.

## Experiment

In this experiment, we compared the classification accuracy when predicting sudden changes in closing prices using only features obtained from Binance data (or Twitter data) with those obtained using both features. In what follows, we call “Twitter features” the features from tweet data, including both linguistic and tweet-specific features, and “Binance features” those from Binance data.

We used conventional performance measures for evaluation, such as accuracy, recall, precision, and macro F1 values.

### Comparison With and Without Twitter Features

The data collection period was from June 29, 2021, to January 6, 2022. Table 2 presents the results of the experiment when the ratio of training data to total data was varied as 70%, 80%, and 90%.

**Table 4:** Results by Twitter features

Class	Precision	Recall	F1-score
Sudden drop	00.0	0.00	0.00
Normal	56.8	99.8	72.4
Sudden rise	44.3	0.70	1.30
Micro average			56.8
Macro average	33.7	33.5	24.6

**Table 5:** Results by binance features

Class	Precision	Recall	F1-score
Sudden drop	30.0	0.100	0.300
Normal	57.1	99.6	72.5
Sudden rise	46.2	1.80	3.50
Micro average			56.9
Macro average	44.4	33.8	25.4

**Table 6:** Results by twitter features $+$ binance features

Class	Precision	Recall	F1-score
Sudden drop	35.4	5.00	8.70
Normal	58.1	96.9	72.6
Sudden rise	38.3	4.00	7.30
Micro average			56.9
Macro average	43.9	35.3	29.6

The baseline in Table 2 is the percentage of correct responses when all the outputs of the prediction are made as normal labels. As can be observed in Table 2, the overall macro F1 value is larger when using Twitter features than when using Binance features alone, with the largest increase being 4.2 percentage points (the difference between the last row and the second last row of Macro F1 values in Table 2, i.e., $29.6 - 25.4 = 4.2$). In addition, as the proportion of training data increases, the prediction accuracy improves significantly more with Twitter features than with Binance features alone.

Tables 4, 5, and 6 list the precision and recall for each label for the divided dataset when the macro F1 value increased by 4.2 percentage points in Table 2. In particular, Table 4 indicates that the model based only on Twitter features could not predict sudden drops. As for Tables 5 and 6, we can observe that the recall of the sudden rise and drop labels is greater with Twitter features, indicating that fewer labels are missed.

However, the precision of the sudden increase in Table 6 is smaller than that in Table 5. This implies that false positives of sudden rise labels are more likely to be detected when Twitter features are used.

### Discussion

**Fig. 4:**
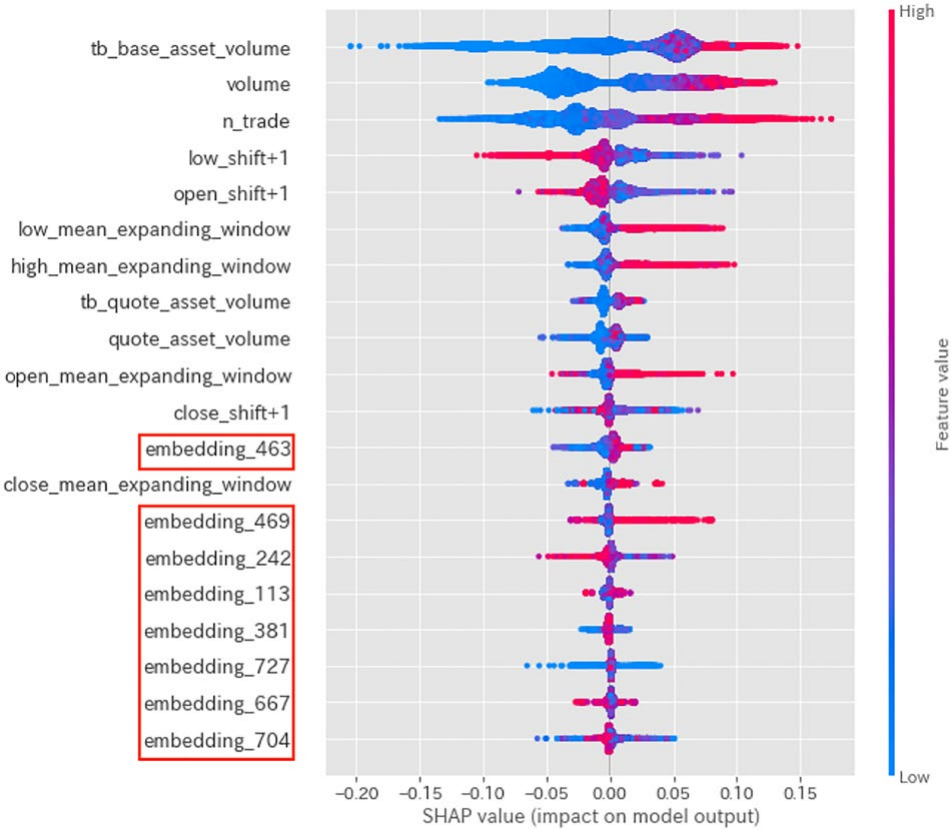
SHAP values for the sudden drop label

**Fig. 5:**
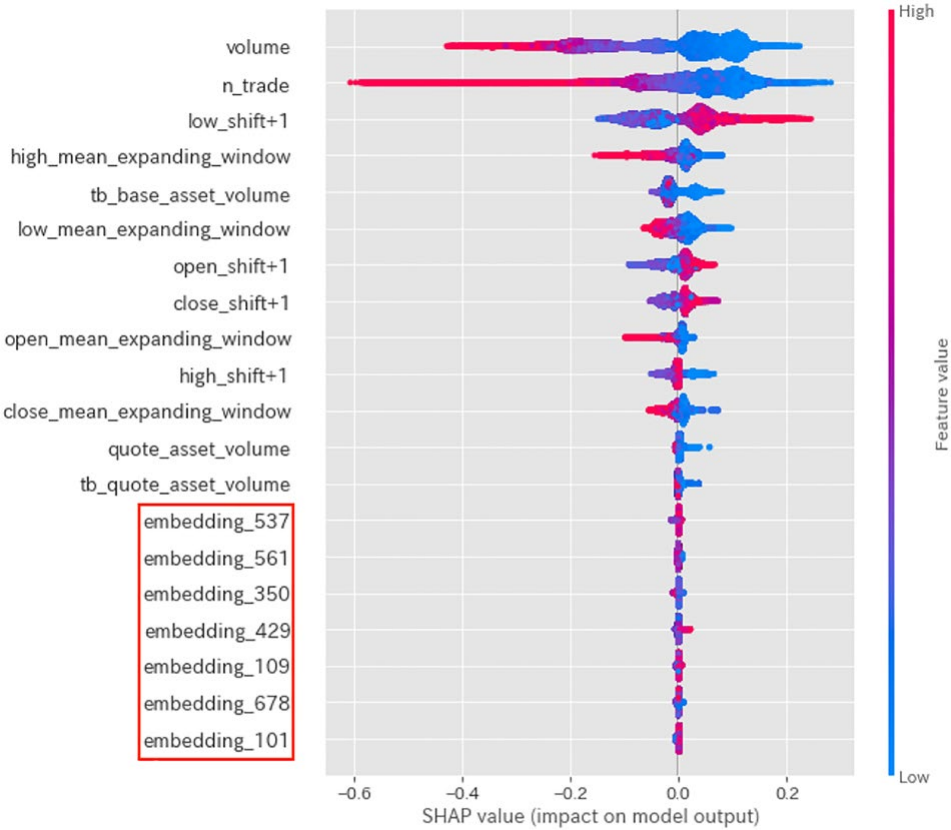
SHAP values for the normal label

**Fig. 6:**
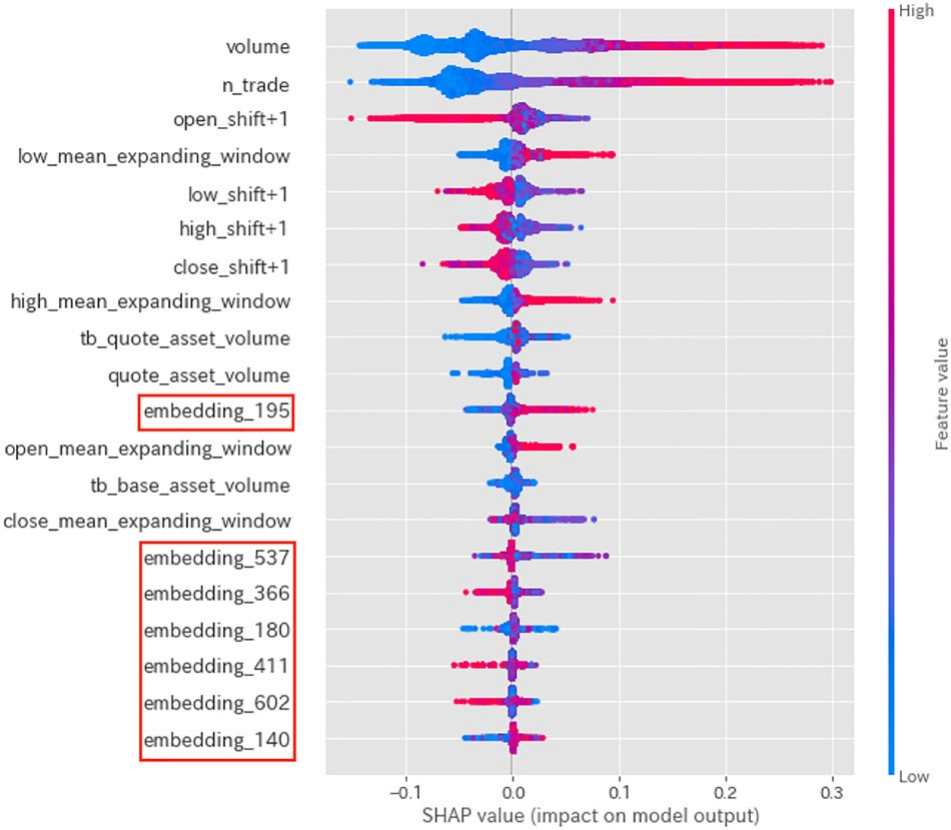
SHAP values for the sudden rise label

Figures 4, 5, and 6 show graphical representations of the SHAP values [6] for the sudden drop, normal, and sudden rise labels, respectively, calculated from our model. Intuitively, SHAP values indicate the predictive power of each feature. In these figures, the magnitude of the value is indicated by color, with larger values indicated by red shading. Using these graphs, we can see how the proposed features function.

The SHAP values displayed in Fig. 5 indicate that Twitter features are unimportant factors when predicting normal labels. Conversely, Figs. 4 and 6 indicate that linguistic features from tweet data become important factors when predicting sudden rise or drop labels. This implies that Twitter features (in particular, linguistic features from tweet data) are helpful in predicting sudden price-changing labels.

In addition, we conducted experiments based on the following assumptions. For the purpose of this study, it is preferable to predict sudden changes in closing prices rather than normal trends.

Tables 4, 5 and 6 indicate that combining Twitter features with Binance features as auxiliary information improves the accuracy, recall, and macro F1 value of sudden changes. However, the use of Twitter features also leads to reduced precision in predicting a sudden rise, which increases the likelihood of false positives. Combining the insight from the discussion about SHAP values in Figs. 4 and 6, we may claim that using linguistic features from tweet data to predict sudden changes in closing price has more advantages than using only Binance features.

## Conclusion

### Summary

In this study, we used a recent machine learning approach to predict sudden changes in the closing price of virtual currencies based on features created from natural language information in social media. First, the embedding of sentences in tweets was created from natural language expressions on social media using Sentence-BERT. The dataset was then created by labeling based on the change in the closing price of the virtual currency and aggregating the embedding created by Sentence-BERT. Finally, we compared the change in the predicted closing price with the presence or absence of the features proposed in this study using the LightGBM.

As a result, Tables 2, 4, 5 and 6 indicate that the macro F1 value was up to 4.2 percentage points larger when we used both Twitter and Binance features.

Figures 4, 5, and 6, which visualize the SHAP values for each label of the prediction by the trained model, show that using both linguistic features from tweet data and Binance features may be useful in predicting sudden price-changing labels.

### Future work

In this study, we used the free version of Twitter API. Therefore, we were only able to acquire data for the previous seven days, which implies that we could only create datasets for a short period and could not change the words in the dataset during the process. Therefore, we could only validate the training and prediction of a localized dataset. It is also necessary to investigate whether a similar trend exists when using a dataset with a wider period. Thus, it is necessary to select the words to be collected from Twitter. In addition, in real-world applications, it is possible to increase the unit size of the time bar and use a more powerful trainer or an ensemble of trainers that perform feature engineering. The following issues will be focused on in future work:

- Revisit the aggregation method of variance expressions that can be created from Sentence-BERT.
- Collect data over a wider period and conduct experiments to determine whether they can be used as features.
- Reconsider the words in the dictionary to be collected and limit the training data to their most recent values.
- Set the time bar to an interval of 1 h instead of 1 min.
- Perform feature engineering using UMAP and aggregate features.
- Use a time-series-aware learner, such as LSTM, so that the learner can also consider the time series.
- Approximate or replace current features with realtime features.

## References

[1] Reimers Nils, Gurevych Iryna (2019) Sentence-Bert: Sentence embeddings using SIAMESE Bert-networks. arXiv preprint arXiv:1908.10084

[2] Devlin Jacob, Chang Ming-Wei, Lee Kenton, Toutanova Kristina (2018) BERT: Pre-training of deep bidirectional transformers for language understanding. arXiv preprint arXiv:1810.04805

[3] Ke Guolin, Meng Qi, Finley Thomas, Wang Taifeng, Chen Wei, Ma Weidong, Ye Qiwei, Liu Tie-Yan (2017). LightGBM: A highly efficient gradient boosting decision tree. Adv Neural Info Process Syst.

[4] Caron Matthew, Muller Oliver (2020) Hardening soft information: A transformer-based approach to forecasting stock return volatility. In *2020 IEEE International Conference on Big Data (Big Data)*, pages 4383–4391. IEEE

[5] Jacques Ohana Jean, Steve Ohana, Eric Benhamou, David Saltiel, Beatrice Guez (2021). Explainable AI (XAI) models applied to the multi-agent environment of financial markets. International Workshop on Explainable.

[6] Lundberg Scott M, Lee Su-In (2017) A unified approach to interpreting model predictions. In *Proceedings of the 31st international conference on neural information processing systems*, pages 4768–4777

[7] Mehta Pooja, Pandya Sharnil, Kotecha Ketan (2021). Harvesting social media sentiment analysis to enhance stock market prediction using deep learning. PeerJ Computer Science.

[8] Microsoft (2017) Light GBM[Source code]. https://github.com/microsoft/LightGBM

